# Do Nitrogen and Phosphorus Additions Affect Nitrogen Fixation Associated with Tropical Mosses?

**DOI:** 10.3390/plants12071443

**Published:** 2023-03-24

**Authors:** Lina Avila Clasen, Aya Permin, Aline B. Horwath, Daniel B. Metcalfe, Kathrin Rousk

**Affiliations:** 1 Department of Biology, University of Copenhagen, Universitetsparken 15, 2100 Copenhagen, Denmarkkathrin.rousk@bio.ku.dk (K.R.); 2 Biological and Environmental Sciences, Faculty of Natural Sciences, University of Stirling, Stirling FK9 4LA, UK; 3 Department of Physical Geography and Ecosystem Science, Lund University, 221 00 Lund, Sweden; 4 Department of Ecology and Environmental Science, University of Umeå, 907 36 Umeå, Sweden

**Keywords:** cyanobacteria, ecosystem ecology, global change, mosses, nitrogen fixation, nutrient limitation, phosphorus, tropical cloud forest

## Abstract

Tropical cloud forests are characterized by abundant and biodiverse mosses which grow epiphytically as well as on the ground. Nitrogen (N)-fixing cyanobacteria live in association with most mosses, and contribute greatly to the N pool via biological nitrogen fixation (BNF). However, the availability of nutrients, especially N and phosphorus (P), can influence BNF rates drastically. To evaluate the effects of increased N and P availability on BNF in mosses, we conducted a laboratory experiment where we added N and P, in isolation and combined, to three mosses (*Campylopus* sp., *Dicranum* sp. and *Thuidium peruvianum)* collected from a cloud forest in Peru. Our results show that N addition almost completely inhibited BNF within a day, whereas P addition caused variable results across moss species. Low N_2_ fixation rates were observed in *Campylopus* sp. across the experiment. BNF in *Dicranum* sp. was decreased by all nutrients, while P additions seemed to promote BNF in *T. peruvianum*. Hence, each of the three mosses contributes distinctively to the ecosystem N pool depending on nutrient availability. Moreover, increased N input will likely significantly decrease BNF associated with mosses also in tropical cloud forests, thereby limiting N input to these ecosystems via the moss-cyanobacteria pathway.

## 1. Introduction

Biological nitrogen fixation (BNF) is a complex process performed by diazotroph organisms that are able to fix atmospheric nitrogen (N_2_) gas to a usable form (ammonia) for assimilation by plants and the ecosystem [1,2,3]. These organisms are often found in association with bryophytes, including mosses, and can significantly contribute to global biogeochemical cycles [4]. In pristine ecosystems, the N input through BNF is a significant source, with previous studies showing that N_2_-fixing cyanobacteria living in association with mosses can contribute 50% to new N input in boreal forests and subarctic ecosystems [3,5,6,7,8,9]. However, it is still unclear if, and to what degree, mosses and associated cyanobacteria are playing a similar role in other pristine ecosystems, such as tropical cloud forests. This is despite the fact that mosses, a major group of non-vascular plants, are highly abundant in tropical cloud forests, and are considered to play an essential role in regulating water fluxes and carbon sequestration within these unique ecosystems [10,11,12,13].

Moss-associated N_2_ fixation rates are influenced by changes in a wide range of abiotic factors, such as moisture and temperature, which are considered to be two of the main factors regulating BNF in mosses. For instance, low moisture reduces N_2_ fixation in mosses [14,15,16], while increasing temperatures can promote it [17]. However, studies have also shown negative effects of elevated temperatures, explained by faster drying and desiccation of mosses, as a result of high temperatures [18]. Tropical cloud forests are ecosystems with high relative humidity and can maintain a fairly stable temperature within the cloud forest cover [19]. Hence, mosses and associated cyanobacteria in those forests experience near-optimal climate conditions to sustain BNF, and moisture should not limit BNF in mosses here, in contrast to other ecosystems, such as arctic tundra [16]. Indeed, a recent study from a tropical cloud forest showed that several moss species host N_2_-fixing cyanobacteria, with relatively high N_2_ fixation activity [20].

Because N_2_-fixing microbial communities associated with mosses are not moisture-limited in tropical cloud forests, nutrient availability is likely a key factor controlling BNF in these ecosystems [19]. However, most studies on BNF and nutrient limitation have focused on vascular plants, with only a few considering soil [21,22,23,24,25], litter [22,23,25] and bryophytes [22,24]. Hence, the importance of nutrient availability in driving moss-associated N_2_ fixation in these highly diverse ecosystems remains largely unexplored, even though they can greatly contribute to the total N budget of tropical regions [12,20] and could control changes in N availability at species and ecosystem levels [3,17,21,26,27].

Increased N availability commonly inhibits activity of the nitrogenase enzyme (responsible for the reduction of N_2_ to NH_3_) [21,26,28]. For instance, a study investigating the effects of nutrient additions on tropical soils found that N additions caused a ~75% decrease in nitrogenase activity [21]. Yet, the rate of N addition, N type and the moss species also matter. For example, Wang et al. [28] applied different rates of inorganic and organic N to two mosses (*Pleurozium schreberi* and *Sphagnum campillifolium*) and found variable results, where inhibition of nitrogenase activity was more prevalent in one species (*P. schreberi*), and only when high doses where applied (10, 20 kg N ha^−1^ yr^−1^). The same study [28] has also found a negative link between nitrogenase activity and pH after N additions. Lower nitrogenase activity in a third subarctic moss (*Hylocomium splendens*) was also linked to low pH levels (ca. < 5.0) in another study [29], suggesting that BNF associated with mosses could also vary according to pH levels, which are modulated by N availability.

In contrast to N, other macronutrients, such as phosphorus (P), can promote BNF, likely due to the fact that the energetically demanding N_2_ fixation process requires P for adenosine triphosphate production (ATP), and for cell metabolism [26,30]. However, P additions to subarctic mosses had variable effects on associated N_2_ fixation activity, with shifts in effects over time. For instance, Rousk et al. [31] and Rousk and Rousk [27] have found no effect or a negative effect of P additions on nitrogenase activity in the common moss *Hylocomium splendens* at high P additions levels. In contrast, when P was added at low concentrations, positive effects on nitrogenase activity were observed after several weeks and months towards the end of the growing season [27,31]. In tropical cloud forests, where relative humidity is high and therefore nutrient limitation can play a significant role for BNF, relatively few studies have assessed P limitation, and the scant available data are far from consistent. For instance, Reed et al. [23] found strong P limitation of N_2_ fixation in soil, litter and leaves, whereas Wong et al. [25] found no P limitation of N_2_ fixation in soil or litter. Responses to N and P additions combined are often not considered, although one study found that the inhibitory effect of available N on N_2_ fixation may be alleviated when P is added together with N [32]. Hence, there is no conclusive evidence yet for P limitation of N_2_ fixation in tropical systems, and there is very little data on nutrient limitation of moss-associated N_2_ fixation in tropical systems.

To address these knowledge gaps, we assessed the effects of N and P additions, in isolation and combined, on N_2_ fixation in three moss species (*Campylopus* sp., *Dicranum* sp. and *Thuidium peruvianum*) collected from a tropical cloud forest in Peru. We hypothesized that (H1) N additions would inhibit N_2_ fixation, (H2) P additions would promote N_2_ fixation, and that (H3) a combined addition of N + P would also reduce N_2_ fixation activity, although with a less pronounced negative effect than adding N alone. We also evaluated if the response of N_2_ fixation to nutrient additions changed over time, since (H4) P could promote N_2_ fixation after several weeks. In addition, we discuss the possible links between cyanobacterial colonization and pH effects on N_2_ fixation since they have been shown to affect N_2_ fixation.

## 2. Results

**Figure 1:**
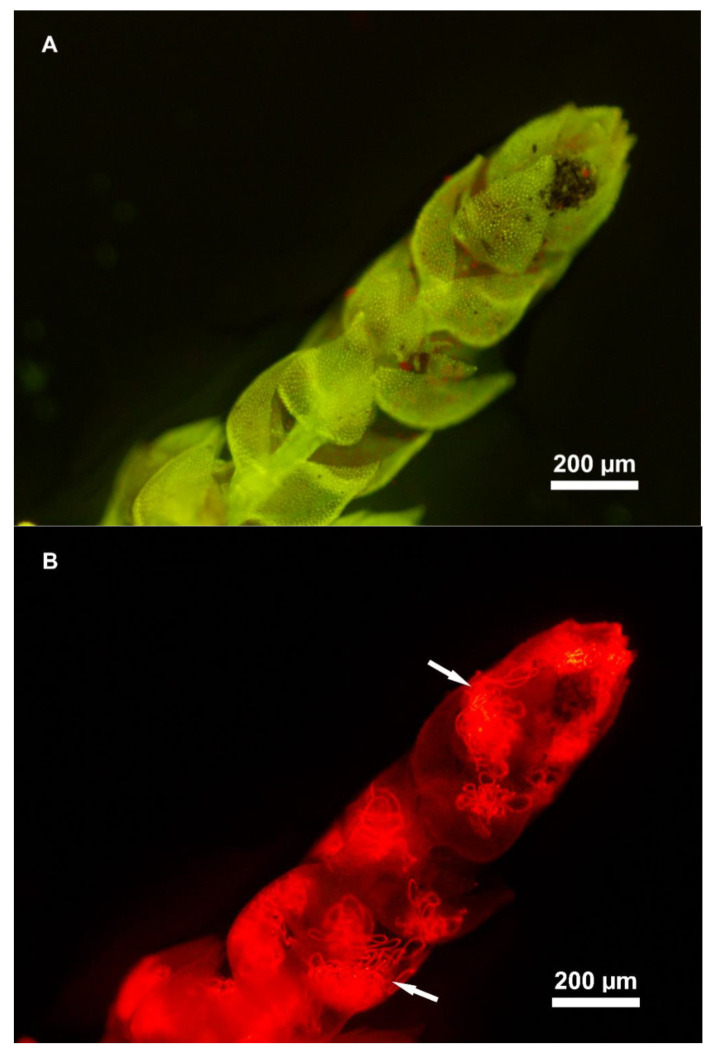
Epifluorescence microscopy image of one shoot of *Thuidium peruvianum* moss collected in a tropical montane cloud forest in south-eastern Peru (40× magnification—Olympus BX61), image with a blue filter (**A**) and image with a green filter (**B**), note the epiphytic cyanobacteria filaments (arrows) in association with the moss shoot. Images: L. A. Clasen.

Cyanobacterial presence was confirmed in all moss species with an epifluorescence microscope. Even though cyanobacterial colonies were observed in all species, they were more abundant in *Thuidium peruvianum*, followed by *Dicranum* sp., and only few sparse colonies were seen in *Campylopus* sp. Only *T. peruvianum* is shown here for visualization (Figure 1).

**Figure 2:**
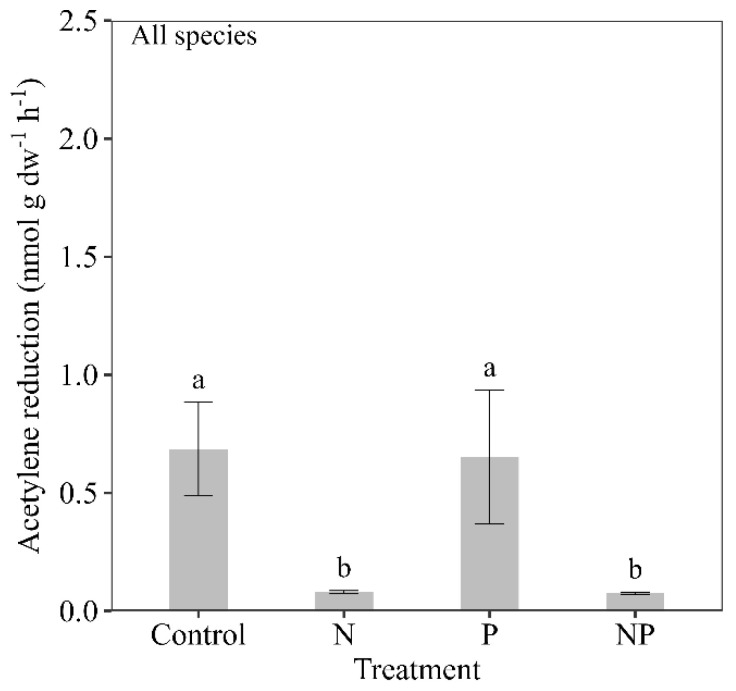
Mean nitrogenase activity (acetylene reduction in nmol g dw^−1^ h^−1^, ±SE) across all time points in response to the nutrient additions (control, nitrogen (N), phosphorus (P) and nitrogen and phosphorus (NP)) across three moss species (*Campylopus* sp., *Dicranum* sp. and *Thuidium peruvianum*) collected in a tropical montane cloud forest in south-eastern Peru. Different letters (a/b) show statistically significant (Tukey HSD test) differences between treatments. *n*=18 (three moss species, six replicates per treatment per moss species).

The treatments that contained N (N and NP) resulted in a significant reduction of nitrogenase activity for all three tropical mosses studied here (*p* < 0.0001, F = 8.56) (Figure 2), with mean nitrogenase activity almost 10 times lower in the N and NP addition treatments (0.08 ± 0.01 for both treatments) compared to the controls (0.69 ± 0.25) and P-only (0.65 ± 0.25) treatments (Figure 2).

**Figure 3:**
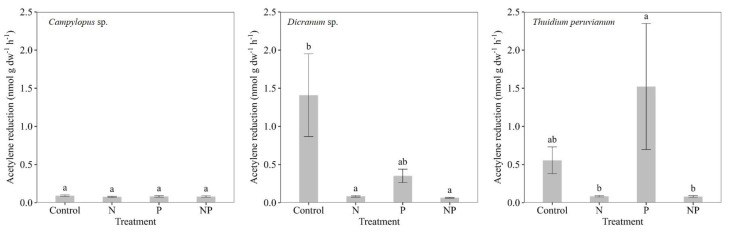
Mean nitrogenase activity (acetylene reduction in nmol g dw^−1^ h^−1^; ±SE, n = 24, across all time points) of the whole experiment in response to nutrient additions (control, nitrogen (N), phosphorus (P) and nitrogen and phosphorus (NP)) in three moss species (*Campylopus* sp., *Dicranum* sp. and *Thuidium peruvianum*) collected in a tropical montane cloud forest in south-eastern Peru. Different letters (a/b/ab) show statistically significant (Tukey HSD test) differences between treatments.

While nitrogenase activity in *Campylopus* sp. did not show significant differences between treatments, nitrogenase activity associated with *Dicranum* sp. had higher activity in the control treatments (1.41 nmol g dw^−1^ h^−1^) compared to the treatments that contained N and NP (*p* < 0.0001). Nitrogenase activity in *T. peruvianum* was higher in the samples that received P compared to samples that received N and NP additions (*p* = 0.03), however high variability among the results was observed (Figure 3). Within the control treatment, the moss with the highest mean rates was *Dicranum* sp. (1.41 ± 0.54) followed by *T. peruvianum* (0.56 ± 0.18) and *Campylopus* sp. (0.09 ± 0.01), which showed very low rates for all treatments and time points. The positive effects of P additions were more pronounced in *T. peruvianum* (1.52 ± 0.83), followed by *Dicranum* sp. (0.35 ± 0.09), but did not statistically differ from the controls due to high variability between replicates. Mean nitrogenase activity for the NP treatment were all low (0.07 ± 0.01, 0.08 ± 0.01 and 0.08 ± 0.01, for *Dicranum* sp., *Campylopus* sp. and *T. peruvianum*, respectively). Treatments containing N-only showed the same low mean nitrogenase activity for all three tropical mosses (0.08 ± 0.01) (Figure 3).

**Figure 4:**
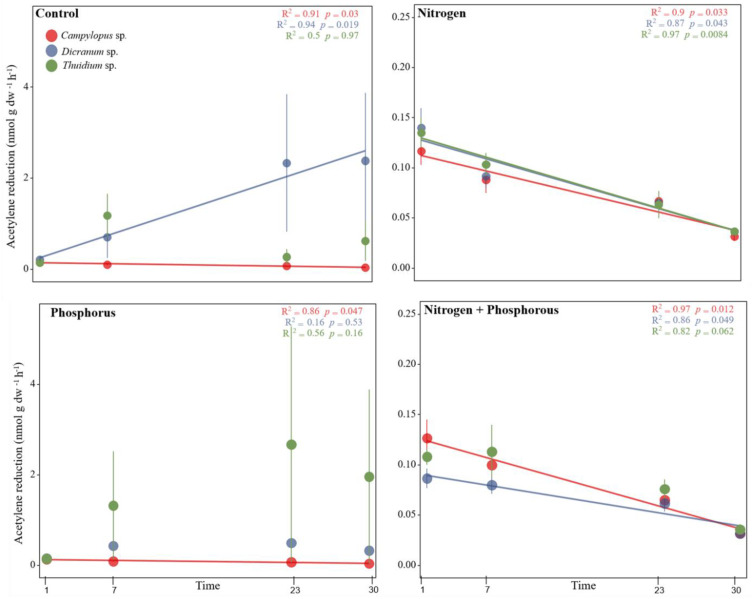
Mean nitrogenase activity (acetylene reduction in nmol g dw^-1^ h^-1^ ±SE, n = 6) over time (1, 7, 23 and 30 days after nutrient additions) in response to nutrient additions (control, nitrogen, phosphorus and nitrogen plus phosphorus) in three moss species (*Campylopus* sp., *Dicranum* sp. and *Thuidium peruvianum*) collected in a tropical mountain cloud forest in Peru. R^2^ and *p* values are given. Trendlines show significant results (*p* < 0.05). Note the different scales at the axis.

Nitrogenase activity was assessed four times over the period of a month to account for longer term nutrient effects but there were no clear trends among all treatments (Figure 4). Nonetheless, a rapid next day response in nitrogenase activity was seen as soon as N was applied, lowering and almost totally inhibiting N_2_ fixation for all species studied here. However, for the control and P treatments, high variability across replicates was observed in *Dicranum* sp. and *T. peruvianum.* Nitrogenase activity increased over time in the control samples in *Dicranum* sp. (*p* = 0.019). *Campylopus* sp. showed low nitrogenase activity over time for all applied treatments (Figure 4).

**Figure 5:**
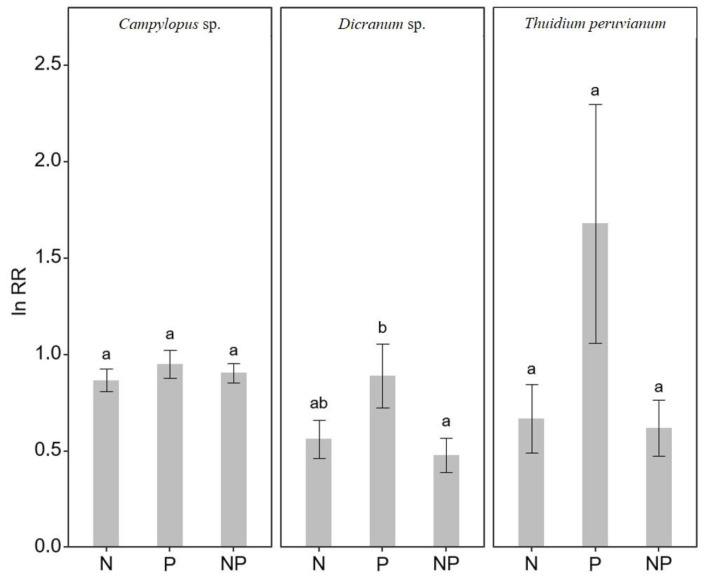
Mean response ratio (ln (natural logarithm) RR, n = 24, ±SE, across all time points) of nitrogenase activity across the treatments (nitrogen (N), nitrogen and phosphorus (NP) and phosphorus (P)) relative to the control treatment, in three moss species (*Campylopus* sp., *Dicranum* sp. and *Thuidium peruvianum*) collected in a tropical mountain cloud forest in Peru. Different letters (a/b/ab) show statistically significant (Tukey HSD test) differences between treatments.

To highlight nutrient addition effects, we calculated the mean response ratios relative to the control treatment, where values above 1.0 suggest promotion and below 1.0 suggest inhibition in nitrogenase activity. The response ratios only indicate promotion in nitrogenase activity after P additions in *T. peruvianum* (1.68 ± 0.62; *p* = 0.135), although this result was not significant, likely due to high variability present within replicates (Figure 5). The response ratio of nitrogenase activity in the P additions was higher than in the NP treatments for *Dicranum* sp. (*p* = 0.031). The response ratios in *Campylopus* sp. were not significantly different between treatments (Figure 5).

**Figure 6:**
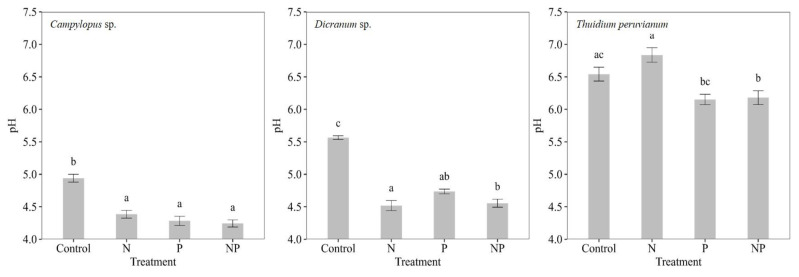
Mean pH values for each moss species (*Campylopus* sp., *Dicranum* sp. and *Thuidium peruvianum*) after nutrient additions (control, nitrogen (N), nitrogen and phosphorus (NP) and phosphorus (P)). Different letters (a/b/ab/ac/bc/c) show statistically significant (Tukey test) differences between treatments.

The mosses’ pH was significantly different between moss species (*p* < 0.0001, F = 82.63) and between treatments (*p* < 0.0001, F = 11.84), with the control treatments showing averages of 4.82 ± 0.13 for *Campylopus* sp., 5.54 ± 0.06 for *Dicranum* sp. and 6.28 ± 0.23 for *T. peruvianum*. The nutrient additions lowered the moss pH in two out of three moss species. For *Campylopus* sp. and *Dicranum* sp., the pH was reduced by all nutrient additions compared to the controls (*p* < 0.0001, F = 13.18 and *p* < 0.0001, F = 19.93, respectively). *Thuidium peruvianum* followed a different pattern where treatments that received P lowered the pH but not N-only additions (*p* < 0.0002, F = 7.24; Figure 6). Nitrogenase activity in *Dicranum* sp. and *T. peruvianum* was highest at pH levels ranging between 5.5 and 6.0.

## 3. Discussion

### 3.1. Nutrient Addition Effects on Moss-Associated N_2_ Fixation

We observed significant nutrient addition effects on nitrogenase activity for two out of the three moss species (*Dicranum* sp. and *Thuidium peruvianum*), where N clearly reduced nitrogenase activity (N_2_ fixation) performed by cyanobacteria living in association with these tropical mosses. This confirms previous studies that used different rates of N additions to test the sensitivity of N_2_ fixation under controlled conditions. For instance, Gundale et al. [14] found a significant decrease in N_2_ fixation associated with *Pleurozium schreberi*, a common feather-moss in boreal forests, with a lower N addition rate (3 kg N ha^−1^yr^−1^) similar to the N deposition recorded at their study site of 2 kg N ha^−1^yr^−1^. In a more extreme N addition study, Rousk and Michelsen [17] tested four different rates (5, 20, 80, 320 kg N ha^−1^ yr^−1^) to another highly abundant moss species in northern regions, *Hylocomium splendens*, and also found negative effects for all rates of N added, except for the lowest rate. Moreover, Wang et al. [28] detected reductions in nitrogenase activity in *P. schreberi* from boreal forests only at N addition levels above 10 kg N ha^−1^ yr^−1^. These findings suggest that inhibition of nitrogenase by N occurs when N availability is higher than the N deposition of the study site. However, these previous studies focussed on N addition effects on moss-associated N_2_ fixation in northern ecosystems, and similar studies on tropical mosses are rare.

Our study shows that N additions reduce N_2_ fixation associated with mosses in tropical regions. Our first hypothesis (H1) was thereby confirmed, indicating that moss N_2_ fixation performed in association with cyanobacteria is reduced or totally inhbited when N is readily available. This is in line with the few other studies in tropical regions. For instance, Cusack et al. [22] found a reducing trend when N was applied at rates of 50 kg N ha^−1^ yr^−1^ on mosses, while another study investigating cyanobacterial colonization in the canopy of a tropical forest found that N_2_ fixation was up-regulated by P and molybdenum, but also inhibited by N additions [24]. For our study, we chose the rate of 30 kg N ha^−1^yr^−1^ since tropical regions are thought to have higher and increasing N deposition rates than boreal and subarctic forests. However, measurements of N deposition from the same study site in Peru recently found N deposition rates of only 4.8 kg N ha^−1^yr^−1^ [20]. Hence, our additions may have been at the high end of the N addition spectrum, which seems to have ensured the inhibition of N_2_ fixation in *Dicranum* sp. and *T. peruvianum*. We could not confirm a negative effect for *Campylopus* sp., since this moss had low cyanobacterial colonization, which is the likely reason for the low N_2_ fixation rate in all treatments.

The P additions caused variable results within and across moss species. We initially hypothesized that P would promote N_2_ fixation (H2), since P is an essential macronutrient for metabolic activity and cellular structure, as well as essential for energy production (ATP) [1,30]. However, nitrogenase activity was promoted by P additions only in *T. peruvianum*, although still with high variability among replicates (Figure 4). Two other field studies [27,31] have found no effect or a negative effect of P additions on nitrogenase activity in the common moss *H. splendens* at high P addition levels (400 kg P ha^−1^ yr^−1^) in the subarctic tundra. However, promotion of N_2_ fixation was seen when P was added in lower concentrations (40 kg P ha^−1^ yr^−1^), similar to our addition rates, as well as after several weeks and months after the additions. Rousk et al. [31] suggest this temporal pattern could be due to the photosynthetic enhancement by cyanobacteria and/or moss, which in turn could supply more carbon (C) and thus, more energy to moss associated-N_2_ fixation. This effect may take weeks to months to manifest. Wong et al. [25] reported that the addition of 18.1 kg P ha^−1^ to soil and litter in a lowland tropical forest had a minimal effect on N_2_ fixation. Our addition rates were much higher (30 kg P ha^−1^yr^−1^), which may explain a clearer trend of N_2_ fixation promotion, at least in one of the investigated mosses.

Our variable results for P additions do not clearly answer the current question on whether P is a limiting factor to N_2_ fixation associated with tropical mosses. Barron et al. [21] have suggested that P can have a longer-term (7 years) beneficial effect on N_2_ fixation in soils, and our 30 day-long experiment may not have been long enough to observe a clearer beneficial effect of P additions that could be translated into higher rates of N_2_ fixation in all tropical mosses. Moreover, no effect of P additions on N_2_ fixation in mosses was found several years after the additions, in a study in arctic tundra [27] or in soils [21]. It may be that not all P is immediately available and consumed by N_2_ fixers. Instead, mosses may be more effectively taking up P for growth, which may benefit the cyanobacterial colonizers and N_2_ fixation only in the longer run. Hence, we cannot completely corroborate our fourth hypothesis (H4) due to variable results and species differences, although we have seen a tendency towards P additions promoting N_2_ fixation in *T. peruvianum* over time.

We also hypothesized that N and P additions combined could still lead to a reduction in nitrogenase enzyme activity and consequently lower the N_2_ fixation rates associated with mosses, but that reduction would be less pronounced than in the N-only treatments due to the possible positive effect of P (H3). This hypothesis could not be confirmed, since N_2_ fixation activity in the NP treatments was similarly inhibited in the N-only treatments across the three moss species, indicating that even with added P, the negative effects of N on N_2_ fixation cannot easily be alleviated. In contrast, Matson et al. [32] found the negative effect of N additions was indeed alleviated by P (at a rate of 10 kg P and 50 kg N ha^−1^yr^−1^) for soil N_2_ fixation in a tropical lowland forest. This different outcome may be due to differences in the environment provided by soil as opposed to moss, as well as differences in timing, dosage and the forest ecotype which could have different P needs [33]. Moss P uptake can vary according to nutrient requirements, and could therefore also influence the availability for associated, N_2_-fixing bacteria. Mosses are efficient in capturing nutrients, including N and P, and absorbed nutrients are often transferred to newly-grown segments, which suggests that mosses are key organisms in regulating nutrient pools of ecosystems [34]. Mosses can homeostatically regulate their elemental composition and stoichiometry, allowing for differentiated biochemical niches among species, and strong inter-species differences in element concentrations are found [35], which suggests different species-specific needs of nutrients, which may also be the case for the here-investigated moss species. Further and more comprehensive work among multiple groups of N_2_ fixers in different tropical ecosystems, as well as research investigating the possible competition and transfer of nutrients between mosses and cyanobacteria is required to develop a more robust understanding of overall patterns and drivers in BNF associated with mosses.

### 3.2. Cyanobacterial Colonization and pH

In addition to nutrients and climatic factors, N_2_ fixation activity by cyanobacteria on mosses seems also to be modulated by pH, which is in turn modulated by nutrient availability, and cyanobacterial abundance [29]. Although our visual assessment via fluorescence microscopy confirmed the presence of cyanobacterial colonies, with larger colonization in *Dicranum* sp. and *T. peruvianum,* no obvious treatment differences were seen. Surprisingly, cyanobacterial biomass (assessed with phycocyanin) did not show clear patterns. Nevertheless, a lack of a link between cyanobacterial biomass and nitrogenase activity in mosses was recently found [36], where the authors suggest that this is likely because N_2_ fixation takes place in phycocyanin-free heterocytes, which are not captured with the phycocyanin extraction, and a link between the frequency of heterocytes and N_2_ fixation activity was indeed found. Hence, cyanobacterial biomass does not necessarily need to be linked to nitrogenase activity, since many cells can in fact be inactive, or only a few cells are active [36].

Nutrient additions appear to have affected the pH in different ways across moss species, with no effects, or N, NP and P effects. Reduction in moss pH after ammonium nitrate addition has been seen in other studies in common boreal forest mosses (*H. splendens*, *P. schereberi* and *Sphagnum capitillifolium*) and was linked to reduction in N_2_ fixation rates [28,35]. The authors even suggest that negative effects of N additions are the result of an effect on moss pH, which in our study may be true for *Dicranum* sp., but not for *T. peruvanium*. The different responses in moss pH to nutrient additions in our study may be due to differences in ion buffering capacities across moss species [37]. Taken together, it seems that the ubiquitous negative effect of N on moss-BNF is not due to a decrease in moss pH, but rather a direct inhibition of the nitrogenase enzyme.

## 4. Methods

### 4.1. Sampling Site and Collection

Mosses were collected from a tropical montane cloud forest near Wayqecha Research Station, south-eastern Peru (latitude: 13°2′56″ S, longitude 71°32′13″2 W) in November 2019. The site is located around 3000 m above sea level, with mean annual air temperature of 10.9 °C, relative air humidity of 90.4% (mean values from the Wayqecha meteorological station, year of 2018) and mean annual rainfall of 1776 mm [10,13]. The ground-covering mosses commonly found in this cloud forest were all found on soil, their identification and field approximation of abundance/coverage were: *Campylopus* sp. (localized abundance—common along trails with a high embankment)/70–90%), *Dicranum* sp. (few localized patches/1–3%) and *Thuidium peruvianum* (common/localized abundant/30–50%*)*. Moss that constituted a single replicate (a composite sample of >10 moss shoots) was collected within a 2 m radius and different replicates (n = 6) were collected at least 10 m apart. Samples were shipped dry to the University of Copenhagen and kept in the dark and cold (4 °C) for ca. 12 months before they were used in the experiment. Prior to the nutrient additions, the samples were individually soaked in double-distilled water (ddH_2_O) for 30 min, allowing for full re-hydration. Ten shoots from each sample (ca. 1–3.2 g fresh weight) were handpicked (except for *Campylopus* sp. which were just weighed (ca. 2 g) due to the very small size of the shoots). Samples were then transferred to 20 mL glass vials, and placed open in growth chambers with 12 h of light at 10 °C (photosynthetically active radiation (PAR) of 250 µmol m^2^ s), and 12 h of darkness at 6 °C—for the entire length of the experiment.

### 4.2. Nutrient Additions

The nutrients were applied to the mosses after five days, allowing for acclimatization to the new conditions. The treatments were added as 1 mL solutions (prepared with double distilled water (ddH_2_O)) and consisted of: (1) nitrogen (N) as NH_4_NO_3_ at a rate of 30 kg N ha^−1^yr^−1^; (2) phosphorus (P) added as NaH_2_PO_4_ · 2H_2_O at a rate of 50 kg P ha^−1^yr^−1^; (3) combined treatment of N + P, same rates as N and P alone; and (4) control treatment (only ddH_2_O added—1 mL). The chosen P addition rate is comparable to the rates used in two studies with subarctic mosses [27,31], and another study with tropical soils and litter [25]. For the N additions, the rate was selected to be higher than the N deposition of the collection site (4.8 kg N ha^−1^yr^−1^), since previous studies showed variable results when the rate is lower or similar to the N deposition, impacted or not by anthropogenic disturbance [5,28,38]. The nutrient effect was assessed over time, with N_2_ fixation measurements taken over a period of one month; 1, 7, 23 and 30 days after nutrient additions. Mosses were kept moist during the experiment by adding 1 mL of ddH_2_O once a week to avoid moisture limiting N_2_ fixation [14,16,18].

### 4.3. Nitrogen Fixation

Nitrogenase activity (N_2_ fixation) was assessed using the acetylene reduction assay (ARA), a commonly used method that relies on the reduction of acetylene (C_2_H_2_) to ethylene (C_2_H_4_) gas, as a measurement of the nitrogenase enzyme [39]. For the ARA incubation, the vials were sealed with a rubber septa and 2 mL of air was replaced, using a syringe, with 2 mL of acetylene gas (corresponding to 10% of acetylene in each glass vial). The incubations started immediately after the nutrient additions with samples kept in the growth chamber for 24 h in the same conditions, as described above. Samples were run on an Agilent 8890 gas chromatograph (GC) coupled to an automatic headspace sampler and equipped with a J&W CarboBOND (column length: 50 m × 0.53 mm × 5 µm), carrier gas: helium, flow rate: 8.4834 mL/min, post run: 1 mL/min, oven temperature: 60 °C, detection method: FID, and pressure set to 10 psi. Acetylene gas (same concentration) was also added to empty vials to account for any residual ethylene presence in the acetylene gas, which was subtracted from the ethylene area of all samples. Extra moss samples were also incubated without acetylene gas to test for natural ethylene production of the moss samples, which was not detected.

### 4.4. Microscopy, pH and Phycocyanin

Following the last nitrogenase activity measurement (30 days), five shoots from each sample were assessed for cyanobacterial colonization using an Olympus BX61 epifluorescence microscope (see Figure 1). Subsequently, moss pH was measured by adding 10 mL of ddH_2_O to the vials, shaken for 30 min and left standing for the same period. With this method, we aimed to measure the environmental pH of the moss surface which resembles the environment the cyanobacterial community living on the moss experiences. Following the pH measurements, the samples were transferred to an oven at 25 °C and left drying overnight. Dry-weight was recorded and the mosses were fine-cut with sterile scissors for follow up analyses.

To estimate the cyanobacterial abundance in association with mosses, we measured the phycocyanin content. Phycocyanin is a photosynthesis pigment that is not produced by mosses, but has been validated for cyanobacterial biomass estimation. Extractions from 0.1 g of moss material were performed following the recommended protocol [40]. Extracts were analyzed using a phosphorimeter-fluorometer, in which fluorescence emission was measured at 643 nm.

### 4.5. Statistical Analyses

Statistical analyses were performed in R (version: 4.2.2. [41]). Nitrogenase activity data were not normally distributed and therefore log-transformed prior to analyses to meet the test requirements. Linear models, one- and two-way ANOVAs were performed to test for the differences in nitrogenase activity among nutrient addition treatments, moss species and time. Phycocyanin and pH values were also tested for correlations with nitrogenase activity. A Tukey HSD test was applied to determine the interaction of the variables and significant differences between treatments, species and time. To address the effect size of the nutrient additions, we calculated the response ratios relative to the control treatment by dividing the treatment values by the control values, in which values above 1.0 suggest promotion, and below 1.0 suggest inhibition of nitrogenase activity, as in Zheng et al. [42].

## 5. Conclusions

The scarcity of knowledge about how nutrient availability affects moss-cyanobacterial associations in tropical ecosystems limits our understanding of tropical biogeochemical cycling now and under future environmental changes. Here, we show that nutrient availability, especially of N and P, can determine how much N enters the ecosystem pool via the moss-associated N_2_ fixation pathway. For instance, if N input is increased—alone or in combination with P—N_2_ fixation will decrease, while P availability can promote N_2_ fixation in some tropical moss species. Hence, the predicted increase in N availability due to global climate change will likely have a negative effect on the ecosystem N input via moss-associated N_2_ fixation, and species-specific responses towards changes in nutrient availability will need to be taken into account when considering global biogeochemical cycles.

## Data Availability

Data will be made available upon request.
